# Left atrial posterior wall isolation using pulsed-field ablation: procedural characteristics, safety, and mid-term outcomes

**DOI:** 10.1007/s10840-023-01728-0

**Published:** 2024-01-05

**Authors:** Patrick Badertscher, Diego Mannhart, Simon Weidlich, Philipp Krisai, Gian Voellmin, Stefan Osswald, Sven Knecht, Christian Sticherling, Michael Kühne

**Affiliations:** 1 Department of Cardiology, University Hospital Basel, Basel, Switzerland; 2 Cardiovascular Research Institute Basel, University Hospital Basel, Petersgraben 4, 4031 Basel, Switzerland

**Keywords:** Atrial fibrillation, Pulsed-field ablation, Posterior wall isolation, Pulmonary vein isolation

## Abstract

**Background:**

Non-pulmonary vein (PV) ablation targets such as posterior wall isolation (PWI) have been tested in patients with persistent atrial fibrillation (AF). Pulsed-field ablation (PFA) offers a novel ablation technology possibly able to overcome the obstacles of incomplete PWI and concerns of damage to adjacent structures compared to thermal energy sources. Our aim was to assess procedural characteristics, safety, and mid-term outcomes of patients undergoing PWI using PFA in a clinical setting.

**Methods:**

Patients undergoing PFA-PVI with PWI were included. First-pass isolation was controlled using a multipolar mapping catheter.

**Results:**

One hundred consecutive patients were included (median age 69 [IQR 63–75] years, 33 females (33%), left atrial size 43 [IQR 39–47] mm, paroxysmal AF 24%). Median procedure time was 66 (IQR 59–77) min, and fluoroscopy time was 11 (8–14) min. PWI using PFA was achieved in 100% of patients with a median of 19 applications (IQR 14–26). There were no major complications. Overall, in 15 patients (15%), recurrent AF/AT was noted during a median follow-up of 144 (94–279) days.

**Conclusions:**

PWI using PFA appears safe and results in high acute isolation rates and high arrhythmia survival during mid-term follow-up. Further randomized trials are essential and warranted.

**Graphical abstract:**

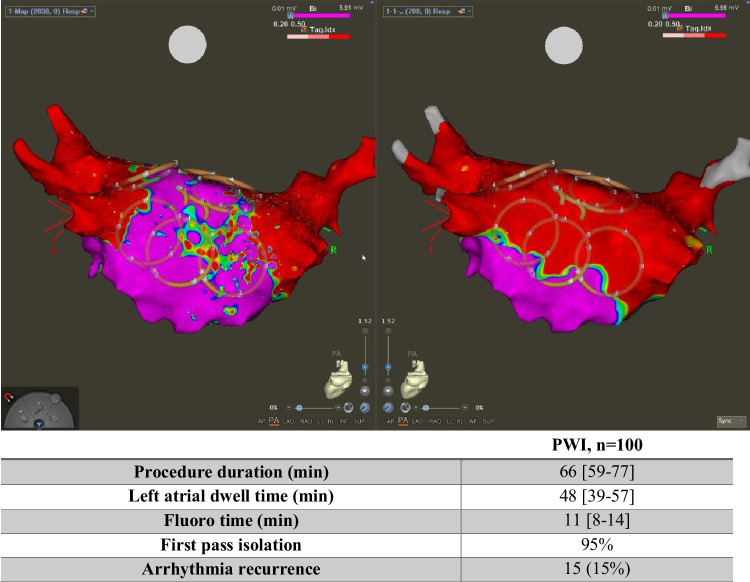

## Introduction

Pulmonary vein isolation (PVI) is an effective treatment option for patients with paroxysmal atrial fibrillation (AF). For patients with persistent AF, long-term success of PVI is limited [1]. It has been increasingly recognized that persistent AF initiation and maintenance of AF may depend on mechanisms outside the pulmonary veins (PV). Therefore, improved ablation strategies and techniques for these patients are needed. Non-pulmonary vein ablation targets such as posterior wall (PW) isolation (PWI) have been tested in patients with persistent AF [2].

Most of the available data on PWI success and complication rates are from observational studies. A 2019 systematic review and meta‐analysis reported an overall single‐procedure 12‐month freedom from atrial arrhythmia of 65% (95% CI, 58–74%) [3]. There is substantial heterogeneity in the distribution of AF subtypes, approaches to perform PWI, intraprocedural definition of PWI, and additional ablation beyond PVI and PWI. This uncertainty currently extends to where it seems difficult to draw conclusions on the true impact of PWI in the treatment of patients with persistent AF. Whether this is due to limitations of current ablation technologies, mainly radiofrequency ablation, or due to the “wrong” ablation target remains unclear at the moment.

Pulsed-field ablation (PFA) is a novel ablation technology that promises improved efficiency with unique myocardial tissue selectivity and a potentially ideal safety profile for performing PWI [4–6]. Little is known about the use of PFA for PWI. Our aim was to assess procedural characteristics, safety, and mid-term outcomes of PWI using PFA.

## Methods

### Study population

In this retrospective, observational study, we enrolled consecutive adult patients (≥ 18 years) undergoing PVI including PWI using PFA at the University Hospital Basel, Basel, Switzerland. Intracardiac thrombi were ruled out by transesophageal echocardiography before the procedure. Pre-procedural imaging, either by computed tomography or cardiac magnetic resonance imaging, was performed in all patients. Written informed consent was provided by all patients prior to the procedure. The study was approved by the local ethics committees and adhered to the Helsinki Declaration.

### Pulsed Field Ablation System

The FARAPULSE Pulsed Field Ablation System (FARAPULSE, Boston Scientific, Natick, MA, USA) consists of a custom generator that delivers a pulsed electrical waveform over multiple channels (Farastar), a 13-F steerable sheath (Faradrive), and a 12-F over-the-wire PFA catheter (Farawave). The PFA catheter contains five splines, each equipped with four electrodes. It can be configured into two poses: the flower and basket configuration.

### Pulmonary vein isolation

In brief, the PFA 31 mm Farawave© catheter (Boston Scientific, Marlborough, MA, USA) was inserted into the left atrium via a single transseptal puncture and PVI was performed as previously described [6]. After transseptal puncture, a guidewire was used to cannulate the veins, and the device was deployed inside the left atrium. PVI with PFA was performed with four applications in basket configuration and four applications in flower configuration per vein. Between pairs of PFA applications, the catheter was rotated by 30–40° after the first two applications in each configuration.

### Posterior wall isolation

**Fig. 1:**
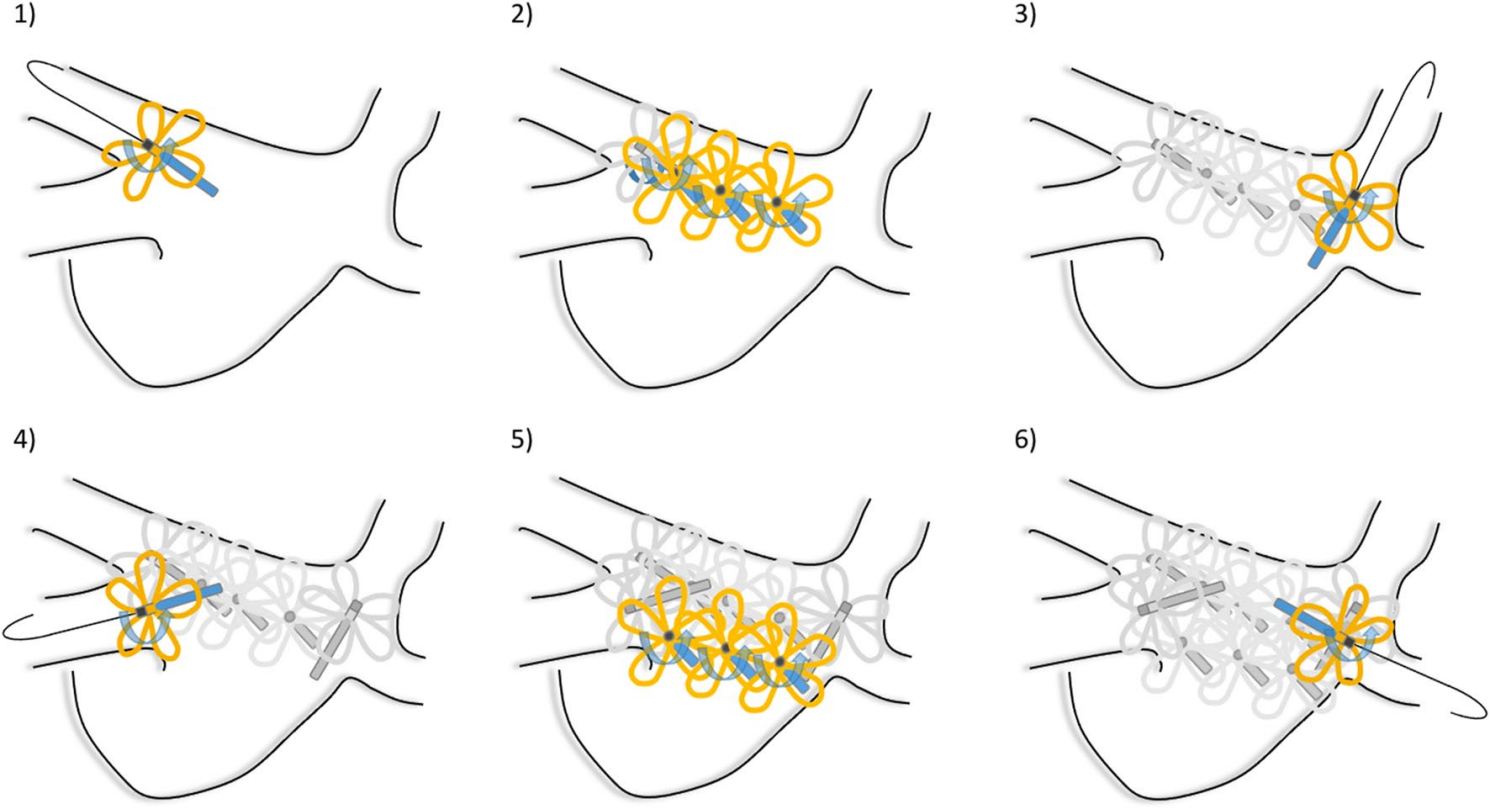
PWI was performed as follows: Using the catheter in flower configuration, two anchor lesions per vein extending to the left atrial posterior wall (PW) were deployed (1, 3, 4, and 6). Then, the wire was pulled back and the PFA catheter was rotated along the entire left atrial PW in a flower configuration in an overlapping fashion (2 and 5)

Indications for PWI were diagnosis of persistent AF and low-voltage areas below a cutoff of 0.2 mV observed after multipolar mapping. PWI was performed as follows: Using the catheter in flower configuration, two anchor lesions per vein extending to the left atrial PW were deployed. Then, the wire was pulled back and the PFA catheter was rotated along the entire left atrial PW in a flower configuration (Fig. 1) with an overlap of one-half of the flower diameter. Per location, two applications were performed with a rotation of 30–40° in between.

### Procedural endpoint

The procedural endpoint was PVI and PWI as assessed at the end of the procedure for all four veins and the PW without a waiting period. For endpoint verification, a three-dimensional electroanatomic mapping (3D-EAM) of the left atrium using a 3D-EAM system (CARTO 3, Biosense Webster, Irvine, CA, USA) in combination with multipolar mapping catheter (Pentaray or Octaray, Biosense Webster) was used. In cases of residual or recovered PV conduction or PW conduction, additional PFA applications were delivered until PVI or PWI was confirmed by repeated PV and PW assessment. First-pass isolation of the PVI and left atrial PW was assessed based on a bipolar voltage cutoff < 0.1 mV.

### Post-ablation management

Oral anticoagulation was continued for at least 2 months. All antiarrhythmic drugs were stopped after the procedure. Major complications were considered. Follow-up was performed at 3, 6, and 12 months after the ablation including history, physical examination, 12-lead electrocardiogram (ECG), 24-h and 7-day Holter ECG, or implantable loop recorder. Repeat ablation procedures were performed using 3D-EAM system in combination with a multipolar mapping catheter. Arrhythmia recurrence was assessed after a 90-day blanking period.

### Statistical analysis

Continuous variables are presented as mean + 1 SD or as median and interquartile range in case of skewed distribution. For continuous variables, comparisons were made using Kruskal–Wallis rank sum or Mann–Whitney *U* test, as appropriate. Discrete variables were compared using Fisher’s exact test. A Kaplan–Meier analysis with a log-rank test was performed to compare the probability of freedom from AF (AF as well as AT) between groups. A *P*-value < 0.01 was considered statistically significant. Analysis was performed using R (R Core Team (2021), R Foundation for Statistical Computing, Vienna, Austria) and RStudio 2023.09.1 (RStudio Team (2019), RStudio, Inc., Boston, MA, USA).

## Results

### Baseline characteristics

Overall, 100 consecutive patients were included (median age [IQR 63–75] years, 33 females (33%), left atrial size 43 [IQR 39–47] mm) between January and November 2023 at a tertiary referral center. Baseline characteristics are summarized in Table 1.

**Table 1:** Baseline characteristics

	PWI, *n* = 100
Age	69 (63–75)
Female sex	33 (33%)
BMI, kg/m^2^	26 (24–31)
Persistent AF	55 (55%)
LA diameter, mm	43 (39–47)
LAVI, ml/m^2^	41 (34–49)
LVEF, %	55 (45–60)
Coronary artery disease	14 (14%)
Diabetes mellitus	8 (8%)
Hypercholesterinemia	31 (38%)
Hypertension	66 (66%)
EHRA score	
I	14 (18%)
IIa	14 (18%)
IIb	20 (25%)
III	23 (29%)
IV	4 (5%)

Abbreviations: *BMI* body mass index, *AF* atrial fibrillation, *LA* left atrium, *LAVI* left atrial volume index, *LVEF* left ventricular ejection fraction, *EHRA* European Heart Rhythm Association

### Procedural characteristics

The median procedure time was 66 (IQR 59–77) min and the median fluoroscopy time was 11 (8–14) min. In 46 patients undergoing first-time ablation, PVI using PFA was achieved with a median of 32 (21–34) applications. In these patients, PWI was performed with a median of 16 (12–20) applications. In 54 patients undergoing redo PVI procedures (after index PVI using radiofrequency energy), 32 patients (59%) demonstrated persistent isolation of all pulmonary veins. In patients undergoing redo PVI, PWI was performed with a median of 23 (16–28) applications. PWI using PFA was achieved in 100% of patients. There were no major complications. We report minor complications in four patients (one patient with AV block after cavotricuspid isthmus ablation, one patient with sinus arrest after electrocardioversion, one patient with bradycardia and sinus arrest, and one patient with a procedure-unrelated airway problem). Procedural characteristics are summarized in Table 2.

**Table 2:** Procedural characteristics

	PWI, *n* = 52
Procedure duration (min)	66 (59–77)
Left atrial dwell time (min)	48 (39–57)
Fluoro time (min)	11 (8–14)
Fluoro dose (Gy cm^2^)	636 (372–1103)
Stick to map time (min)	16 (12–22)
Map time (min)	14 (10–19)
Ablation time (min)	19 (12–30)
Waiting period (min)	10 (6–15)
First-pass isolation	95%
Arrhythmia recurrence	15 (15%)
Median follow-up time (days)	144 (94–279)
AF recurrence	12 (12%)
AT recurrence	3 (3%)

Abbreviations: *AF* atrial fibrillation, *AT* atrial tachycardia

### Arrhythmia-free survival

Overall, in 15 patients (15%), recurrent AF/AT was noted during a median follow-up of 139 (89–274) days. Three patients (3%) presented with AT and 12 patients (12%) with recurrent AF. Recurrent AF/AT was noted in 7 out of 46 patients (15%) undergoing first-time ablation and in 8 out of 54 patients (15%) undergoing repeat ablation.

## Discussion

Our main findings are as follows: First, PWI using PFA was achieved in 100% of patients with a median procedure time of 66 min and a median of 19 applications. Second, first-pass isolation of the left atrial PW was 95% indicating that the proposed workflow of applying two anchor lesions per vein and then rotating the device along the entire left atrial PW is an efficient ablation strategy. Third, no major complications were observed in this series. Fourth, arrhythmia-free survival during mid-term follow-up was 85%.

It is well known for the PW of the left atrium to be arrhythmogenic [7, 8]. Several hypotheses exist: Drivers (such as focal activations and rotors) perpetuating AF are more commonly found in the PW, predominantly at sites of patchy fibrosis, which is prevalent in the PW in patients with AF [7]. Endocardial‐epicardial dissociation has increasingly been postulated to be an important AF mechanism, particularly in persistent AF [8]. The extent of endocardial‐epicardial dissociation is greater in the PW compared to other atrial regions.

Several studies have described the use of the two most common energy sources for PWI: radiofrequency (RF) ablation and cryoballoon ablation [9–15]. The main limitations of both used energy sources include the non-durability of PWI isolation. Using information from remapping from redo procedures during the follow-up period, reconnection rates for the PWI were 66% when using RF [15] and 60% when using cryoballoon [14]. Further studies using invasive remapping during FU to assess the durability of PWI after PFA are warranted.

The more recent POBI-AF study [9] randomized 217 patients to PVI versus PVI with added PWI by RF ablation. Freedom from any documented AF without antiarrhythmic drug during 1-year follow-up was 51% for PVI and 56% after PVI with added PWI and not different among groups. The CAPLA study was a randomized trial that compared PVI versus PVI with added PWI in patients with persistent AF and used RF ablation [1]. The recurrence rate of AF after 1 year was not different among groups (52.4% versus 53.6%). Mean procedural time was 142 (SD, 69) min for the PVI with PWI group with a reconnection rate for the PWI of 69% as assessed in patients undergoing redo procedures.

These studies highlight the limitations of thermal energy sources: incomplete PWI in a significant number of cases, PW and PV reconnection during follow-up, and limitations of energy delivery on the PW because of safety concerns, in particular damage to the adjacent esophagus [16]. Due to its tissue selectivity, these safety concerns can be limited by PFA. Furthermore, in contrast to PWI using a focal RF catheter to create a roof line and posterior wall line, a complete myocardial elimination of the posterior is performed. This might reduce the risk of epicardial reconnection of the posterior wall.

Only a few other studies assessed the use of PFA for PWI [17]. The PersAFOne study assessed the safety and lesion durability of both PVI and PWI with PFA in 25 patients with persistent AF [18]. The acute procedural success of PWI was 100%. No mucosal lesions were observed at post-procedure esophagogastroduodenoscopy in all patients. Invasive remapping 3 months after the index procedure demonstrated durable isolation of all left atrial PWs (100%). Freedom from atrial arrhythmias after 1 year was 92%.

These initial findings show that PFA is an efficient modality to perform PWI safely and quickly lowers the bar to perform PWI in addition to PVI in patients with persistent AF. Since the PW adds little to atrial contraction, no negative adverse impact of PWI can be expected. However, the question of high arrhythmia-free survival needs to be confirmed in larger randomized studies, such as the PIFPAF-PFA study. This study will randomize patients towards PVI versus PVI and PWI using PFA. Randomization will be performed after the assessment of the scar via 3D-EAM.

We acknowledge several limitations in this study. First, this was a single-center, prospective non-randomized study with all its limitations. Second, we did not use uniformly implantable loop recorders in all patients, which is currently considered the gold standard for rhythm monitoring in clinical trials after AF ablation [19, 20]. Third, since PFA is relatively new and with currently only one commercially available catheter, the optimal number of applications is not known. Further refinement might potentially improve the effectiveness of PFA for PWI. Fourth, PVI and PWI were assessed via 3D-EAM system, but not with additional testing of the absence of pace capture at maximum pacing output (> 10 mV) from the PW.

In conclusion, PFA seems to allow PWI in an efficient fashion resulting in high arrhythmia-free survival during mid-term follow-up. Further randomized studies are warranted.

## Data Availability

The datasets generated during and/or analyzed during the current study are available from the corresponding author on reasonable request.
